# PROTOCOL: Family treatment drug courts for improving parental legal and psychosocial outcomes

**DOI:** 10.1002/cl2.1024

**Published:** 2019-08-14

**Authors:** Suzanna Fay, Elizabeth Eggins

**Affiliations:** ^1^ Department of Humanities and Social Sciences, School of Social Sciences University of Queensland Brisbane Queensland Australia; ^2^ Department of Health, School of Applied Psychology Griffith University Brisbane Queensland Australia

## 1 BACKGROUND

### 1.1 The problem, condition or issue

A substantial portion of parents involved in child welfare systems have co‐occurring substance abuse issues (Laslett, Room, Dietze, & Ferris, 2012; Miller, Orellana, Johnson, Krase, & Anderson‐Nathe, 2013; Williams, Tonmyr, Jack, Fallon, & MacMillan, 2011; Young, Boles, & Otero, 2007). In addition, child welfare cases characterized by parental substance abuse tend to result in more detrimental outcomes for families, than cases without parental substance abuse issues (Brook & McDonald, 2009; Connell, Bergeron, Katz, Saunders, & Tebes, 2007; Smith, Johnson, Pears, Fisher, & DeGarmo, 2007). In the United States, the high rate of parental substance abuse amongst child welfare cases transitioned to judicial settings (Miller, 2004) led to adaptation of the adult drug court model into a Family Treatment Drug Court to deal with child welfare cases characterized by parental substance abuse. Since their 1994 inception in the United States (US), Family Treatment Drug Courts (FTDCs) have increased in popularity over the last 15 years (Bruns, Pullmann, Weathers, Wirschem, & Murphy, 2012; Fay‐Ramirez, 2015). For example, US FTDCs have increased from 2 in 1994 to 495 in 2018 (Lemus & Ritcher, 2018) and the model has been recently implemented in the United Kingdom (Bambrough, Shaw, & Kershaw, 2014; Harwin et al., 2018) and Australia (Children's Court of Victoria, 2019; Marshall, 2015). This trend is, in part, driven by increasing recognition that individuals that come through the criminal, juvenile and civil (family) court system often have co‐occurring issues such as mental health or addiction issues that need to be addressed in order to motivate behavior change in the interest of children, or the parent themselves (Fay‐Ramirez, 2015; Tiger, 2012). For child welfare cases, parental substance abuse is seen as the problem that hinders the establishment of a stable family environment that would enable the child's return to parents’ care. Thus, the primary goal of FTDCs is to treat the parental legal and psychosocial issues in child welfare cases, with a key aim being to reunify families, achieve permanent placements for children in a timely manner, and address substance abuse issues among parents (Gifford, Eldred, Vernerey, & Sloan, 2014).

Although FTDCs are becoming more popular around the world, the state of evidence for the impact of FTDCs on parent‐level outcomes, beyond parent–child reunification, is currently unclear. Some research suggests that the FTDC model promotes better long‐term outcomes for parents and families over their traditional courtroom counterparts (e.g., Picard‐Fritsche, Bryan, Kralstein, & Farley, 2011). However, other research is less equivocal. For example, Lloyd (2015) argues that parents are less successful within the FTDC model and appear to be at higher risk of having their parental rights terminated than if their case is processed in a traditional dependency court. In addition, scholars have raised concern over whether studies showing positive outcomes have been driven by the FTDC therapeutic model of justice, or the extent of surveillance used by the FDTCs to monitor parents and families (Marlowe, DeMatteo, & Festinger, 2003; Tiger, 2012). Moreover, existing reviews of FTDCs lack methodological rigor, fail to integrate the full range of parental legal and psychosocial outcomes, or tend to focus on child outcomes (see “Existing Reviews”). Although a focus on child outcomes is warranted, changes in parent‐level factors are critically important for generating changes in child outcomes. Without a comprehensive understanding of the effect of FTDCs on parental legal and psychosocial outcomes, the full impact of FTDCs on vulnerable families with both child welfare and parental substance abuse issues remains unclear. Therefore, a methodologically rigorous review and synthesis of the growing number of FTDC impact evaluations is required to thoroughly understand whether FTDCs are effective for improving parental legal and psychosocial outcomes.

### 1.2 The intervention

FTDCs are an example of a growing number of “specialized” or “problem orientated” courts that use a nonadversarial approach for substance abusing parents who have ongoing child welfare cases being monitored by the judicial system (Gifford et al., 2014; Lloyd, 2015; Picard‐Fritsche et al., 2011). The model originated in the United States in 1994 and sit within the civil jurisdiction, whereas in other countries, FTDCs sit within the family court system (e.g., Australia and United Kingdom (Levine, 2012).^1^ The primary goal of FTDCs is to reunite children with their parents as well as achieve parental sobriety by using (a) an extended treatment team of mental health practitioners, child advocates, attorneys and social workers; (b) regular in‐ and out‐patient drug/alcohol treatment; (c) regular drug and alcohol testing; and (d) regular court hearings to maintain supervision by the treatment team (Edwards & Ray, 2005; Haack, Alemi, Nemes, & Cohen, 2005; Chuang, Moore, Barrett, & Young, 2012).

**Figure 1:**
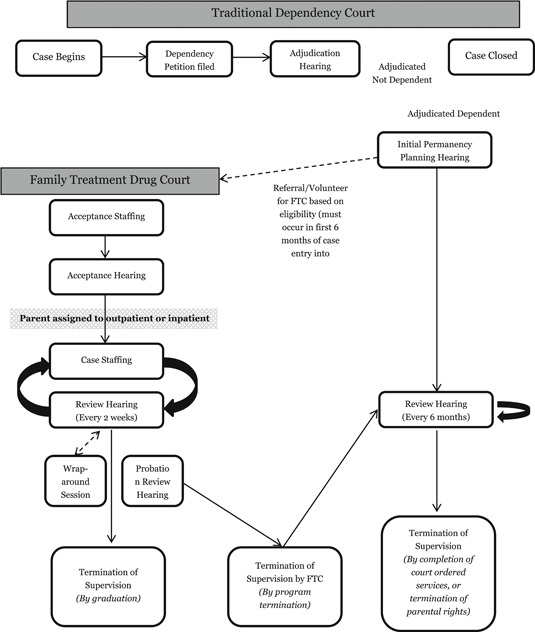
Case processing for Family Treatment Drug Courts (FTDCs) and traditional Dependency Courts

FTDCs differ from a traditional pathway in a number of ways (often referred to as Traditional Dependency Court). Traditional dependency^2^ courts have jurisdiction for all child protection matters regardless of whether the parent has co‐occurring substance abuse issues. Core differences between the FTDC model and the traditional child welfare model, involve increased monitoring in FTDC (as often as weekly) versus traditional court (every 6 months), increased contact with a treatment team in FTDCs, and increased involvement of the treatment team in case management and inpatient/outpatient substance abuse treatment (Fay‐Ramirez, 2015; Gifford et al., 2014; Picard‐Fritsche et al., 2011). Figure 1 shows typical FTDC processes in comparison to typical traditional court pathways and Appendix A provides a description of common graduation and components of an 18‐month FTDC program.

FTDCs typically involve in‐ or out‐patient treatment of drug and alcohol addiction, monitoring and supervision whist in the FTDC program, incentives and sanctions for treatment compliance and other prosocial family behavior, mental health counseling and parenting assistance (Fay‐Ramirez, 2015; Harden, Harper, & Popovits, 2018). In addition, FTDCs often use a range of consequences in order to maintain compliance with FTDC orders such as—at the most extreme end—criminal sanctions or limited parental rights to a child (Fay‐Ramirez, 2015; Harden, Harper, & Popovits, 2018).

Existing reviews and qualitative descriptions of FTDCs show a high degree of consistency in their description of the intervention (Ashford, 2004; Fay‐Ramirez, 2015; Worcel, Furrer, Green, Burrus, & Finigan, 2008). Parents with child protection concerns and co‐occurring substance abuse issues can volunteer for an FTDC program. Once accepted into the FTDC, parents typically begin a series of assessments that determine what kinds of resources parents and their children may need. This will include inpatient or outpatient treatment, but also a wide range of other services to help improve the family environment, from employment assistance to parenting training. Parents will come to court every two weeks for a “review hearing” where the judge and treatment team will review the parents’ progress and make decisions about problems or issues that arise for parents and their children. These review hearings serve as an opportunity for the treatment team, the judge and the parent to interact about each case. Review hearings continue through the parents’ time in FTDC until they “graduate” from the program having met all program requirements. It is also a key model component that the treatment team meet informally outside of court to solve problems that arise and discuss each case prior to court review hearings. This integrated courtroom model is typically (a) nonadversarial, (b) makes decisions on a case‐by‐case basis although it relies on a systematic method of applying behavioral modification techniques and (c) focuses on rehabilitation rather than punishment (Fay‐Ramirez, 2015).

FTDCs involve scaffolded program goals with incentives for good behavior and less supervision over time. FTDC programs vary in length but typically last for 18‐months. This 18‐month time frame is usually dictated by legislation, which determines how much time the court has to find a permanent placement for the child who is subject of the child protection concern. For example, US federal policy dictates that a reunification hearing is necessary at 12 months and termination of parental rights if a child is in foster care for 15 out of 22 months. This placement can be with the parent or through adoption or legal guardianship (previously long‐term foster care). Graduation requirements from FTDCs most often consist of a range of goals having been reached by the parent. These goals include a period of sobriety from substance abuse, consistent engagement with counseling or other support services, suitable housing for the family, employment or other goals determined on a case‐by‐case basis by the FTDC treatment team over the course of the program (Fay‐Ramirez, 2015). However, graduation requirements are not entirely subjective and are described in FDTC participant manuals.

### 1.3 How the intervention might work

The FTDC intervention is based on the recognition and understanding that the majority of cases in the child protection system involve multi‐layered and complex issues (Gifford et al., 2014). The FTDC is thought to (a) reunify families and (b) improve substance abuse issues by integrating treatment, social support and social resources into a nonadversarial, rehabilitation‐based court program. These options for substance abuse treatment and other support services are offered in the traditional courtroom model, however, compliance with court mandated treatment or resources is often low and considered to contribute to the high number of families with repeated contact with the child protection system (Fay‐Ramirez, 2015). FTDCs are thought to improve compliance by using the judge and treatment team to monitor and compel compliance.

Although FTDCs, and Drug Courts more broadly, precede the concept of Therapeutic Jurisprudence (TJ), TJ has been used to understand and justify FTDCs (Fay‐Ramirez, 2016; Nolan, 2009). In its simplest terms, TJ is the understanding that the law and experiences of the law can have potentially therapeutic or anti‐therapeutic effects (Wexler & Winick, 1991). In the case of FTDCs, the nonadversarial nature of the program, the case‐by‐case decision making, and the understanding that the focus should be on rehabilitation are thought to underpin increased compliance with mandated court orders involving treatment and services.

Designed to offer more rehabilitative pathways to prosocial behavior for offenders with co‐occurring drug and/or mental health issues, the drug court model (including FTDCs) deviate from the traditional courtroom model by offering more discretion for judges, flexible treatment options, and increased monitoring of the offender (Levine, 2012). Drug Courts, including FTDCs, are thought to provide increased contact and individualized treatment to offenders in order to maximize positive behavioral outcomes, including desistence from crime, family reunification, sobriety and mental health stability/treatment.

Goldkamp, White, and Robinson (2001) provide a framework for understanding how Drug Courts (including FDTCs) increase positive outcomes for its clientele.

**Figure 2:**
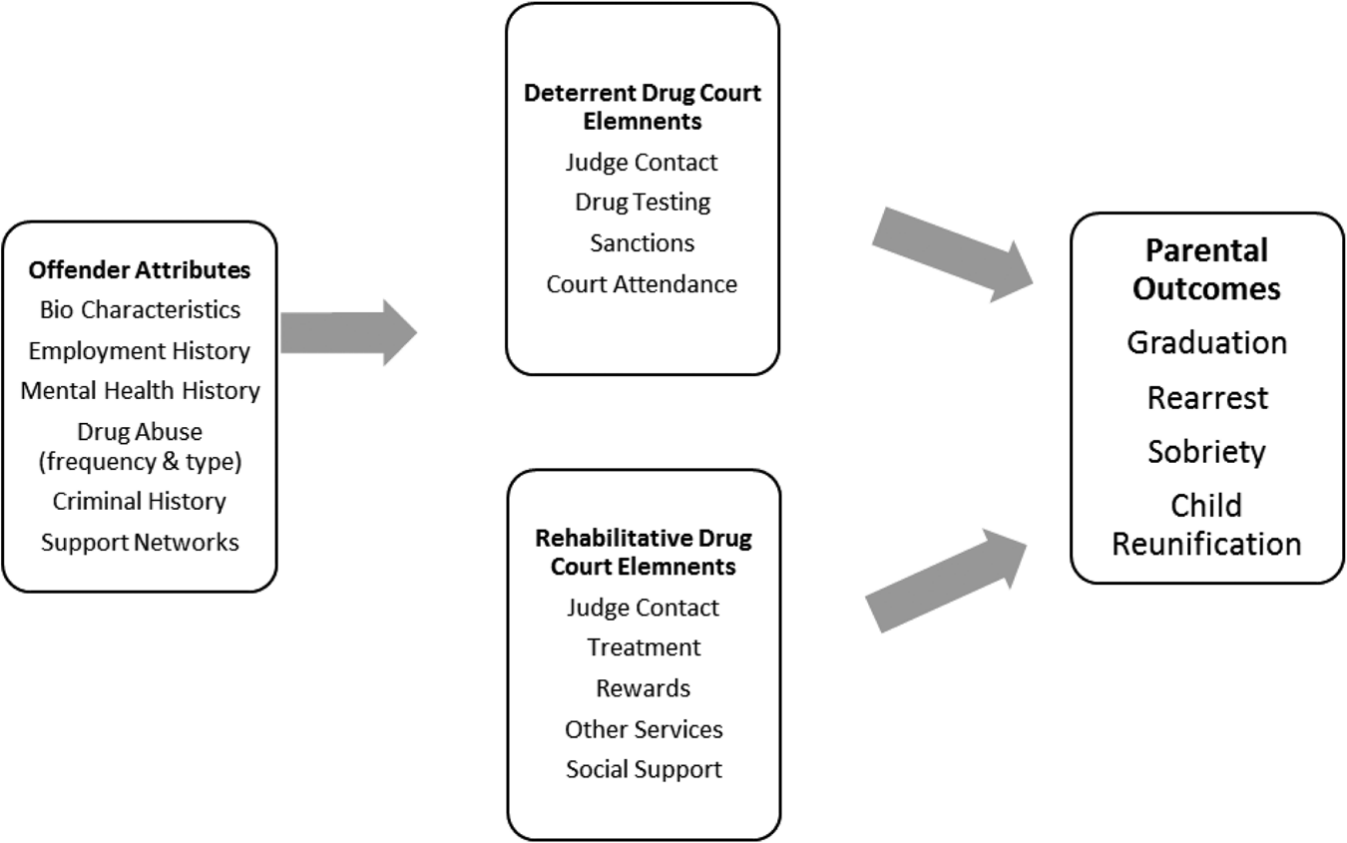
Family Treatment Drug Court logic model

Figure 2 provides a logic model that describes the characteristics of the Drug Court mode that help promote positive outcomes including sobriety, mental health stability, desistance from crime and resolution existing criminal justice warrants/cases, and family reunification. Offender attributes often determine whether parents/offenders agree to FTDC/Drug Court supervision, but they also shape parental outcomes, mental health issues, employment opportunities, available support networks and history of drug abuse and treatment, which all relate to increasing likelihood of reoccurring contact with the criminal justice system and the child welfare system (Miller et al., 2013). Goldkamp et al. (2001) describe Drug Court characteristics as (a) deterrent; and (b) rehabilitative, reflecting the need to provide incentive to submit to the Drug Court program, and provide flexible services matched to the individual needs of the parent/offender. Deterrent drug court elements include increased monitoring well above what would be expected in a traditional court model. This typically includes increased contact with the supervising judge, frequent drug testing, required and frequent court attendance, and sanctions when program rules are violated. Rehabilitative elements of drug courts include frequent contact with the judge (builds rapport), drug and mental health treatment, opportunities to access other services such as parenting classes, educational services, and use of the courtroom workgroup as social support—a core element of the therapeutic approach. Parents and offenders are also provided with rewards for good behavior and reaching sobriety milestones. These deterrent and rehabilitative elements of the drug court together are thought to produce more positive outcomes for offenders/families.

### 1.4 Why it is important to do the review

There are several existing reviews that either focus entirely FTDC or capture one or more FTDC impact evaluation studies. Lloyd (2015) conducted a systematic review to examine the impact of FTDCs on children's time in foster care and reunification and conducted a qualitative synthesis of FTDC evaluations that used research designs with a comparison group. Wittouck, Dekkers, De Ruyver, Vanderplasschen, and Vander Laenen (2013) aimed to synthesize existing evidence for drug treatment courts on substance use more generally and provide a qualitative synthesis that includes FTDC studies. Eldred and Gifford (2016) used systematic search and screening to identify peer‐reviewed US research that examines the use of legal approaches for addressing child maltreatment. The authors provide a brief narrative synthesis with cites to some existing FTDC evaluation studies. Other authors provide traditional narrative reviews of FTDC literature that capture studies assessing the impact of FTDCs on a range of different child and parent outcomes (Choi, 2012; Harrell & Goodman, 1999; Marlowe & Carey, 2012).

Lloyd (2015) and Zhang, Huang, Wu, Li, and Liu (2019) provide the most methodologically robust reviews, yet these reviews still either only focus on child outcomes or the synthesis only includes studies that reported statistically significant outcomes (Lloyd, 2015) or a restricted timeframe (Zhang et al., 2019). While Wittouck et al. (2013) and Eldred and Gifford (2016) include some studies with parent‐level outcomes, their reviews are subject to bias by only searching one database (Wittouck et al., 2013) or excluding research not published in peer‐review outlets (Eldred & Gifford, 2016). Moreover, although remaining narrative reviews are useful for gauging the breadth and characteristics of existing Family Treatment Drug Court impact evaluations, their synthesis approach is inadequate for providing reliable and precise estimates of intervention impact (Borenstein, Hedges, Higgins, & Rothstein, 2009; Littell, Corcoran, & Pillai, 2008).

These methodological quality issues and gaps in content coverage limit the conclusions that can be drawn about the effectiveness of Family Treatment Drug Courts for improving parental legal and psychosocial outcomes. Therefore, this review will both update and enhance the existing body of reviews by providing a synthesis of the evidence for the effectiveness of Family Treatment Drug Courts on legal and psychosocial outcomes for parents. This is important for explicating and providing an unbiased assessment of the *overall* effectiveness of FTDCs. Contingent on the extant evidence, this review may also enable examination of the specific components or conditions that moderate the effectiveness of FTDCs for parental legal and psychosocial outcomes. As such, this review will provide practitioners and policy makers with a comprehensive and robust summary of the extant evaluation evidence for FTDCs to inform their future decision‐making.

## 2 OBJECTIVES

The overarching objective of this review is to systematically gather and synthesize the extant impact evaluation literature relating to Family Treatment Drug Courts. Specifically, this review will address the following research questions

- What is the impact of Family Treatment Drugs Courts on parental legal and psychosocial outcomes?
- Does the impact of Family Treatment Drug Courts vary according to the (a) jurisdiction; (b) type of outcome measure; (c) type and/or severity of substance abuse; (d) number of treatment components; or (e) length of treatment?

## 3 METHODOLOGY

### 3.1 Criteria for including and excluding studies

#### 3.1.1 Types of study designs

Studies will be eligible for the review if they report on a quantitative impact evaluation of a Family Treatment Drug Court, using eligible participants and parent‐level legal or psychosocial outcome measures. In addition, the impact evaluation must employ a randomized experimental design or methodologically robust quasi‐experimental design with an eligible comparison condition. Eligible comparison conditions are placebo, no treatment, waitlist control, treatment‐as‐usual and alternative treatment.

When defining an inclusion threshold for nonrandomized studies in systematic reviews, some research synthesists recommend that inclusion thresholds should be based on the design features of studies rather than traditional research design labels (e.g., Higgins et al., 2013; Reeves, Deeks, Higgins, & Wells, 2011). The rationale for this approach is based on the (a) the argument that risk of bias will affect specific design features versus an overall research design category; and (b) the disparity and possible ambiguity across disciplines in regards to research design terminology. For the purposes of this review, methodologically robust quasi‐experimental designs are defined as those that permit causal inference by minimizing threats to internal validity. Examples of “robust” quasi‐experimental designs include those that maximize treatment and comparison group equivalence through matching (e.g., propensity score matching), reduce maturation threats by measuring outcomes at multiple time points pre‐ and postintervention (e.g., interrupted time‐series, cohort panel designs), or adjust for confounding factors through statistical modeling (e.g., multiple regression, multilevel modeling). Single group studies with one pre‐intervention and one postintervention outcome measure will be excluded from the review due to high threats to internal validity.

To be included in the meta‐analyses, studies must report data that is sufficient to calculate a standardized effect size. If the data report in eligible studies is insufficient, the document authors will be contacted to obtain the required data.

#### 3.1.2 Types of participants

This review will focus on families who have transitioned to a FTDC program due to the co‐occurrence of child welfare and parental substance abuse issues. This situation is often referred to as a child dependency/welfare case, which is defined as an open case where child protective agencies have asked the court to intervene with the family due to child welfare concerns. These cases may or may not include out‐of‐home child placement (Gifford et al., 2014; Lloyd, 2015; Picard‐Fritsche et al., 2011). Given the focus on parental outcomes, the primary research participants in eligible impact evaluations must be either substance abusing parent(s) or entire families characterized by parental substance abuse issues. A family is defined as at least one child and one parent. A parent is defined as an individual who is responsible for providing emotional, physical and/or financial care of a child. This definition includes teenage, biological, foster, adoptive, or kinship caregivers. A child is defined as an individual between of 0–18 years who is under the care of at least one parent.

Families are only eligible for FTDC programs if they are classified as having parental substance abuse and child welfare issues, so we will not define specific evidence thresholds to substantiate the presence of substance abuse or child welfare issues. We will include abuse of alcohol, illicit and/or prescription drugs.

#### 3.1.3 Types of interventions

Interventions to be included in this review are those that evaluate a FTDC. We will include studies where the authors label the intervention as a FTDC. Where the authors do not use this explicit terminology, we will assess the intervention and include the study if the intervention aligns with descriptions of the FTDC used in the existing FTDC literature. Specifically, to be classified as a FTDC, the intervention must (a) utilize a nonadversarial courtroom approach to child protection cases where the parent(s) has a co‐occurring substance abuse issue; and (b) require participants to participate in drug treatment and/or a range of other resources to help stabilize the family environment and facilitate parent‐child reunification. Studies will be included in the review if they include placebo, no treatment, waitlist control, treatment‐as‐usual and alternative treatment comparison conditions (e.g., cases treated by the traditional court process, often referred to as “dependency court”).

#### 3.1.4 Types of outcome measures

This review will include a range of parent‐focused legal and psychosocial outcomes, however, conceptually distinct outcomes will be analysed separately. Outcomes will be included if they are comprised of official data or are measured using standardized or nonstandardized instruments (e.g., diagnostic, observation or self‐report modalities).

FTDCs are a judicial intervention, so we envision judicially focused outcomes will be the most common legal outcomes reported in primary studies. Examples of judicially focused outcomes include specific orders served (e.g., injunctions and warrants), statutory orders relating to parenting (e.g., termination of parental rights and specific parenting orders), and degree of compliance with judicial orders (e.g., failing drug tests, failure to complete treatment). However, if eligible studies also report legal outcomes pertaining to the law enforcement and correctional arms of the criminal justice system, they will be included in the review. Possible examples of additional legal outcomes include arrests, convictions or sentencing data (e.g., type and length of sentence).

One of the primary aims of the FTDC model is to address both the substance abuse and psychosocial issues that have resulted in the family becoming involved in the judicial system. Therefore, psychosocial outcomes will be included in this review to provide a comprehensive synthesis of FTDC effectiveness. However, delineating the exact nature of these outcomes is difficult due to the individualized nature of the treatment provided to families. Specifically, the approach taken in each case can vary depending on the underlying issues for the family, which can range from the need for inpatient substance abuse treatment to wider issues around housing or employment. Therefore, we tentatively define eligible psychosocial outcomes to include those relating to level of substance abuse (alcohol and other substances) and outcomes important for providing a stable and nurturing family environment (e.g., positive parenting behaviors, housing status and employment). We will also code cost‐effectiveness and treatment acceptability (e.g., participant perspectives of the intervention) outcomes if they are reported in eligible studies.

#### 3.1.5 Duration of follow‐up

Studies will be included in this review regardless of the length of follow‐up after the intervention. However, where the length of follow‐up varies across studies, we will group and synthesize studies according to similar follow‐up durations. For example, short (e.g., 0–3 months post intervention), medium (>3 months, <6 months) and long‐term follow‐up (>6 months post intervention).

#### 3.1.6 Types of settings

To be eligible for inclusion in the review, the primary study setting must be within the court system, however, given the nature of FTDCs, we will include studies where the intervention includes the court system and other formal and informal social support systems. Although most FTDC research has occurred in the United States, we will include studies from all countries.

#### 3.1.7 Additional eligibility criteria

Studies that satisfy the abovementioned eligibility criteria will be included in the review regardless of publication status. While documents written in languages other than English will not be excluded from the review, they will only be assessed for final eligibility and included in the syntheses if a translation can be sourced.

### 3.2 Search strategy

During a piloting phase, it was found that the search below provided the optimum balance between sensitivity and specificity. Due to the small number of search results captured in the pilot and the variation that was found in abstracts across disciplines, the systematic search for the review will utilize a broad search that uses only intervention search terms.

#### 3.2.1 Search terms

Wherever possible, the following search string will be applied to the Title, Abstract, Keywords and indexing fields of search locations so that documents will be captured if the title, abstract or keywords contain one or more of the following search terms:

“family drug court*” OR “family treatment drug court*” OR “family drug treatment court*” OR (family N15 “drug court*”) OR “dependency drug court*” OR “dependency court*”

Due the unique search operators indexing systems across electronic databases, the exact search will need to be tailored to each location and may vary slightly across search locations. Where the functionality of a search location does not permit complex search strategies, a simplified version of the search will be utilized. The search will place no limits on publication date, document language, or publication status. However, clearly ineligible document types will be excluded from search results if the specific search location permits (e.g., book reviews). Each search will be recorded in a search record as per recommended guidelines (see Kugley et al., 2017; Littell et al., 2008; see Appendix B).

#### 3.2.2 Search locations

The systematic search will be conducted across multiple disciplines and source types in order to reduce the potential for disciplinary and publication bias (see Table 1 for search locations). Four additional steps will be undertaken to identify eligible documents not already captured, following completion of the full‐text eligibility screening stage. First, reference harvesting will be conducted on the final corpus of eligible documents and existing narrative reviews. Second, citation tracking will be undertaken for all eligible documents. Third, prominent scholars relevant to the review topic will be personally contacted to enquire about eligible documents not yet published or disseminated. Fourth, the most recent issues of key journals will be hand‐searched to identify potentially eligible documents not yet indexed in academic databases.

**Table 1:** Systematic search locations

**Academic databases**
Campbell Collaboration Library of Systematic Reviews
Cochrane Database of Systematic Reviews
Database of Abstracts of Reviews of Effectiveness (DARE)
Expanded Academic ASAP
HeinOnline (Law Journal Library)
ScienceDirect
Scopus
EBSCO platform:
Criminal Justice Abstracts
Web of Science platform:
Conference Proceedings Index
Current Contents Connect
Medline
Social Science Citation Index
ProQuest platform
Criminal Justice Database
Dissertation and Theses Global
Family Health
Health and Medical Complete
International Bibliography of the Social Sciences
Nursing and Allied Health
Psychology Journals
Research Library
Social Science Database
Sociological Abstracts
Social Services Abstracts
OVID platform
Cumulative Index to Nursing and Allied Health Literature (CINAHL)
Embase
Joanna Briggs Institute EBP Database
PsycINFO
PsycEXTRA (gray literature)
Informit platform
Australian Criminology Database (CINCH)
DRUG
Family and Society Abstracts (FAMILY)
Health and Society
Health Collections
Humanities and Social Sciences Collection
**Gray literature sources and websites**
Alcohol and Alcohol Science Database (ETOH)
Alcohol Concern (UK)
American Institutes for Research
AODstats.org.au
Australian Centre for Child Protection
Australian Institute of Family Studies
Australian Therapeutic Jurisprudence Clearinghouse
Bibliomap
California Evidence‐Based Clearinghouse for Child Welfare
Canadian Research Institute for Social Policy (CRISP)
CareData
CEBC (California Evidence‐Based Clearinghouse for Child Welfare)
Centre for Court Innovation (http://www.courtinnovation.org/topic/drug‐court)
CEBI (Centre for Evidence‐Based Intervention, Oxford University)
Centre for Evidence‐based Public Health Policy
Child Abuse and Neglect Digital Library (canDL)
Child Abuse, Child Welfare & Adoption Database
Child Trends
Child Welfare Information Gateway
ChildData
DART‐Europe E‐theses Portal
Database of Promoting Health Effectiveness Research (DoPHER)
Directory of Open Access Journals
Drug Court Clearinghouse (http://www.american.edu/spa/jpo/initiatives/drug‐court/resources.cfm)
e‐Theses Online Service (eThOS)
Early Intervention Foundation (www.eif.org.uk)
Economic and Social Research Council (ESRC, Regard database)
European Monitoring Centre for Drugs and Drug Addiction (EMCDDA)
Evidence for Policy and Practice Information & Coordinating Centre (EPPI‐Centre)
Family Drug Support Australia (www.fda.org.au)
FLoSse Research
Foundation for Alcohol Research and Education (Australia)
Gray Literature Network Service
Health Technology Assessment Database (HTA)
Intute: Social Science
MDRC (https://www.mdrc.org/publications)
National Association of Drug Court Professionals (http://www.nadcp.org)
National Centre for State Courts (https://www.ncsc.org)
National Centre on Substance Abuse and Child Welfare (https://ncsacw.samhsa.gov/resources/resources‐drug‐courts.aspx)
National Child Traumatic Stress Network
National Criminal Justice Reference Service (NCJRS)
National Drug Court Institute (https://www.ndci.org/about‐ndci/)
National Drug Court Resource Centre (https://ndcrc.org)
National Drug and Alcohol Research Centre (NDARC, Australia)
National Institute on Alcohol Abuse and Alcoholism
National Institute on Drug Abuse (NIDA, US)
NPC Research (http://npcresearch.com/specialty‐areas/)
National Research Register (NRR, National Health Service, UK)
National Society for the Prevention of Cruelty against Children (NSPCC)
National Technical Information Service (NTIS)
Networked Digital Library of Theses and Dissertations
NHS Economic Evaluation Database (EED)
OAIster
OpenDOAR
OpenGrey
Parent Mental Health systematic map database (hosted by EPPI‐Centre)
PubMed
ProjectCork.org
RAND Drug Policy Research Centre (https://www.rand.org/multi/dprc.html)
Register for Open Access Repositories (ROAR)
SAMHSA's National Registry of Evidence‐based Programs and Practices
Save the Children
Social Care Online
Social Care Institute for Excellence (SCIE, including ELSC)
Social Sciences Literature Information System (SOLIS)
Social Science Research Network (SSRN)
The Evidence Network
The Urban Institute
Turning Research into Practice (TRIP database)
Turning Point
What Works Clearinghouse
What Works for Children
United Nations Office on Drugs and Crime (UNODC)
**Trial registries**
Australian and New Zealand Clinical Trials Registry
ClinicalTrials.gov
Clinical Trials Results
Cochrane Central Register of Controlled Trials (CENTRAL)
ISRCTN Registry (controlled‐trials.com)
NIH RePORTER
Trials Register of Promoting Health Interventions (TRoPHI)
Unreported Trials Register
UK Clinical Research Network (UKCRN Study Portfolio)
WHO International Clinical Trials Registry Platform
**Hand searched journals**
Addiction
Child Abuse and Neglect
Child Abuse Review
Child and Adolescent Social Work Journal
Child Maltreatment
Children and Youth Services Review
Crime and Delinquency
International Journal of Therapeutic Jurisprudence
Journal of Drug Issues
Journal of Experimental Criminology
Journal of Social Work Practice
Justice Quarterly
Juvenile and Family Court Journal
Law and Social Inquiry
Substance Abuse

### 3.3 Description of methods used in primary research

Most existing impact evaluations of FTDCs utilize quasi‐experimental designs. For example, Worcel, Furrer, Green, Burrus, and Finigan (2008) used propensity score matching to examine the impact of FTDCs (*n* = 301 families) compared to business‐as‐usual child welfare services (*n* = 1,220 families). Outcomes included reunification, whether participants entered treatment sooner, whether participants spend longer in treatment, and treatment completion. Similar impact evaluations are reported by Bruns et al. (2012) and Lloyd (2015).

### 3.4 Criteria for determination of independent findings

The software that will be used for this review enables nesting of multiple dependent documents pertaining to one study. Should there be dependent studies, all studies will be coded and data will be extracted from the most complete report of the study and the study will only be included once in a meta‐analysis for each conceptually unique outcome. If studies report on multiple conceptually similar outcomes, the effect sizes will be averaged using the method described by Borenstein et al. (2009). If a study utilizes a research design with clustering (e.g., study sites assigned to conditions), the method suggested by Fu et al. (2013) and Higgins and Green (2011) will be used to adjust the standard error (SE). In these cases, where studies do not report the required intra‐class correlation coefficient (ICC), the approach taken by Barlow, Bergman, Kornør, Wei, and Bennett (2016) will implemented to assess the impact of clustering on effect estimates. Specifically, Barlow et al.'s (2016) systematic review of group‐based parenting interventions took the approach of conducting sensitivity analyses to examine whether the results of their meta‐analyses varied with ICCs of 0, 0.03, 0.02 and 0.1.

### 3.5 Details of study coding categories

#### 3.5.1 Title and abstract screening

The initial phase of assessing study eligibility will begin with title and abstract screening all unique records identified by the systematic search. After removing duplicates and ineligible document types (e.g., book reviews, blog posts) from the results of the systematic search, all records will be imported into the review management software, *SysReview* (Higginson & Neville, 2014) Each title and abstract (record) will then be assessed according to the following exclusion criteria

(1) Ineligible document type
(2) Document is not unique
(3) Document is not about Family Treatment Drug Courts.

Although all efforts will be made to remove ineligible document types and duplicates prior to screening, automated and manual cleaning can be less than perfect. As such, the first two exclusion criteria will be used to remove ineligible document types and duplicates prior to screening each record on substantive content relevance.

Records retained at the title and abstract screening stage will progress to literature retrieval, where the full‐text document will be located and attached within *SysReview* before progressing to full‐text eligibility screening. Where full‐text documents cannot be retrieved via existing university resources, they will be ordered through the university libraries of the review authors or by contacting study authors.

#### 3.5.2 Full‐text eligibility screening

The full‐text of each document progressing through the literature retrieval stage will be screened for final eligibility according to the following exclusion criteria

(1) Ineligible document type
(2) Document is not unique
(3) Document is not an FTDC study
(4) Ineligible participants
(5) Ineligible outcome measure
(6) Ineligible research design.

Although all efforts will be made to remove ineligible document types and duplicates in prior stages, these types of records can sometimes progress into later stages, for example, where duplicate records are not adjacent to each other during screening or where screeners cannot unequivocally determine if record is ineligible based on the title and abstract. As such, the first two exclusion criteria will be used to remove ineligible document types and duplicates prior to screening each document for final eligibility.

#### 3.5.3 Full‐text coding and risk of bias assessment

Eligible studies progressing from the full‐text screening stage will be coded within *SysReview*, using the coding companion provided in Appendix C. Broadly, studies will be coded according to the following domains:

(1) General study characteristics (e.g., document type, study location)
(2) Participants (e.g., sample characteristics by condition)
(3) Intervention (e.g., intervention components, intensity, setting)
(4) Outcomes (e.g., conceptualization, mode of measurement, time‐points)
(5) Research methodology (e.g., design, unit and type of assignment)
(6) Effect size data
(7) Risk of bias.

Risk of bias will be evaluated using either the Cochrane randomized or nonrandomized risk of bias tools, whereby studies will be rated across domains as having high, low or unclear risk of bias. Where a domain is rated as “unclear” study authors will be contacted to obtain missing data. Results of the risk of bias assessment will be presented in summary tables and in a risk of bias summary figure. Depending on the data available, sensitivity analysis will be used to examine the impact of risk of bias on effect estimates and corresponding confidence intervals. Possible analyses include: forest plots stratified by level of risk, moderator analysis, or meta‐regression. The degree of variation in risk of bias across included studies will determine the approach taken to incorporate risk of bias in statistical analyses. For example, statistical analysis may be stratified by level of risk or all studies may be included in one analysis with a narrative discussion of the risk of bias (see Higgins & Green, 2011, for more detail).

### 3.6 Statistical procedures and conventions

Statistical analyses will utilize the random effects inverse variance method (Lipsey & Wilson, 2001) and will be performed in R using the *rmeta* program code available at https://CRAN.R‐project.org/package=rmeta (Lumley, 2015). FTDC evaluations typically report binary outcomes (e.g., parental substance abuse relapse: yes/no), and in these cases, effect sizes will be computed as odds ratios. Where outcomes are reported as continuous measures, Hedges *g* (standardized mean differences, SMDs) will be computed and then transformed into odds ratios for meta‐analyses (see, Borenstein, Hedges, Higgins, & Rothstein, 2009). Mean effect sizes will be reported along with their corresponding confidence intervals, both in‐text and in forest plots.

Where studies report multiple points of follow‐up, effect sizes will be calculated for each time‐point, but synthesized separately with studies that have similar outcome time‐points. If component studies report baseline and postintervention outcome data, SMDs will be calculated using baseline adjusted mean differences (i.e., mean change scores) and the change score standard deviations, will be standardized using the raw standard deviation within groups. Where authors do not report the standard deviation for mean change scores, Lipsey and Wilson's (2001) formula will be used to calculate the standard deviation ($\frac{s_{T1+}^{2}s_{T2}^{2}/2}{\sqrt{2(1-r)}}$). If studies report follow‐up outcome data, post‐only outcome data will be used to estimate SMDs, and follow‐up outcomes will be analysed separately from postintervention outcomes.

Heterogeneity of the studies will be examined using the *I* ^2^ statistic, *χ* ^2^ test and *τ* ^2^ (Higgins & Thompson, 2002). Using the variables outlined in the Objectives section, moderator analyses will be used to explore potential sources of heterogeneity. Specifically, the analogue to analysis of variance will be used for categorical moderators and regression‐based approaches will be used for continuous moderators. Depending on the data reported in included studies, additional exploratory subgroup analyses may be performed, however, we will clearly distinguish between a priori and exploratory analyses in our reporting.

Assessment of publication bias will be the final stage of analysis and will first entail inspection of funnel plots for asymmetry to identify whether effect size estimates are influenced by publication bias. If asymmetry is detected, subgroup analyses will be conducted to assess whether effect sizes significantly differ by publication status of the included studies.

### 3.7 Treatment of qualitative research

We do not plan to include qualitative research as part of this review.

## ROLES AND RESPONSIBILITIES

Suzanna Fay‐Ramirez is a comparative criminologist with extensive experience in researching juvenile, adult and family court models (Fay‐Ramirez, 2015) as well as child maltreatment and child welfare responses more broadly. Her research focuses on examining the effectiveness of implementing court programs, such as drug courts, in the United States and Australian context. She has extensive experience and training in high‐level statistical methods for the social sciences, as well as qualitative methods of inquiry, and is a frequent reviewer for systematic review studies and experimental methods.

Elizabeth Eggins has co‐authored and managed a range of research projects grounded in systematic review methodology, including Campbell Collaboration and industry funded systematic reviews, and scoping or qualitative research that uses systematic search, screening, and coding techniques. Her research focuses on vulnerable families more generally, with a particular focus on quantitative impact evaluations and systematic reviews.

- Content: Fay‐Ramirez, Eggins
- Systematic review methods: Eggins, Fay‐Ramirez
- Statistical analysis: Fay‐Ramirez, Eggins
- Information retrieval: Eggins, Fay‐Ramirez.

## SOURCES OF SUPPORT

Elizabeth's contribution to this review has been supported by an Australian Government Research Training Program Scholarship.

## DECLARATIONS OF INTEREST

Suzanna Fay‐Ramirez has published in the area of FTDCs, yet has no investment (financial or otherwise) in the results of individual evaluation studies or the results this review.

## PRELIMINARY TIMEFRAME

We will submit a draft of the final review in January 2019.

## PLANS FOR UPDATING THE REVIEW

Both authors will update this review 3 years after the publication of the first final review.

## AUTHOR DECLARATION

### Authors’ responsibilities

By completing this form, you accept responsibility for preparing, maintaining and updating the review in accordance with Campbell Collaboration policy. The Campbell Collaboration will provide as much support as possible to assist with the preparation of the review.

A draft review must be submitted to the relevant Coordinating Group within 2 years of protocol publication. If drafts are not submitted before the agreed deadlines, or if we are unable to contact you for an extended period, the relevant Coordinating Group has the right to de‐register the title or transfer the title to alternative authors. The Coordinating Group also has the right to de‐register or transfer the title if it does not meet the standards of the Coordinating Group and/or the Campbell Collaboration.

You accept responsibility for maintaining the review in light of new evidence, comments and criticisms, and other developments, and updating the review at least once every five years, or, if requested, transferring responsibility for maintaining the review to others as agreed with the Coordinating Group.

### Publication in the Campbell Library

The support of the Coordinating Group in preparing your review is conditional upon your agreement to publish the protocol, finished review and subsequent updates in the Campbell Library. The Campbell Collaboration places no restrictions on publication of the findings of a Campbell systematic review in a more abbreviated form as a journal article either before or after the publication of the monograph version in Campbell Systematic Reviews. Some journals, however, have restrictions that preclude publication of findings that have been, or will be, reported elsewhere and authors considering publication in such a journal should be aware of possible conflict with publication of the monograph version in Campbell Systematic Reviews. Publication in a journal after publication or in press status in Campbell Systematic Reviews should acknowledge the Campbell version and include a citation to it. Note that systematic reviews published in Campbell Systematic Reviews and co‐registered with the Cochrane Collaboration may have additional requirements or restrictions for co‐publication. Review authors accept responsibility for meeting any co‐publication requirements.

## Footnotes

1 See National Drug Court Institute & Centre for Substance Abuse Treatment (2004) and Levine (2012) for a summary of the difference between FTDCs and the traditional drug court model.

2 The term “dependency” in this case refers to the dependency of the child on the judiciary/government to care for the child when it is determined that the parent cannot. This is the typical term used to refer to child protection matters in the juvenile or civil court systems in the United States. The term dependency does not refer to substance dependency.

## APPENDIX A

### Typical FTDC Eligibility Requirements and Application Process

**To be considered for eligibility**

- Be willing to admit to the court that his/her child is dependent; or have an existing dependency finding on his/her children.
- Be chemically dependent and willing to go to treatment.
- Be 18 years of age or older.
- Be willing to sign a Consent to Release Confidential Information Form so that the team may share information with other team members and outside community providers.
- Have the ability both mentally and physically to fully participate in the program.
- Not be a perpetrator of sexual abuse or felony child abuse.
- Applications/referrals to FTDC must be received no later than six months from the date on which the dependency petition was filed.

**Application Process**

- Participation in FTDC is voluntary and parents with existing dependency cases have the ability to apply to be considered for the FTC program.
- Parents meet with FTDC staff to verify eligibility.
- Eligible parents are referred for an immediate drug and alcohol evaluation.
- Clinical assessment to verify chemical dependency on drugs and/or alcohol.
- FTDC treatment team.^3^ meets to determine eligibility and discuss all available case information for a recommendation to the FTDC Judge.
- FTDC Judge makes final decision on eligibility based on FTDC treatment team recommendation.

**Graduation Requirements**

- 6‐months consecutive clean time.
- Children living at home for 6 months (or in permanent placements).
- Successful discharge from substance abuse treatment program.
- Consistent attendance at support program.^4^ (must be documented)
- Suitable housing arranged (drug free).
- Outstanding warrants resolved and Dependency court services completed.
- Support system, relapse prevention plan and life plan established.

## APPENDIX B

### Search Record Form Template

**Table:**

Name of Database*
Search Date
Database Supplier/Platform
Database Coverage (dates, frequency of updates, content coverage)
Full Search Syntax
Number of search results
Notes (e.g., reason for modification of search)

*Database used here for simplicity, this form will be used for all search locations.

*Note*. Adapted from Kugley et al. (2017).

## APPENDIX C

**Full‐Text Coding Form** 5

### General study details

1. Study ID [*textbox*]
2. Report ID [*textbox*]*
3. What type of document is this study? [*dropdown menu*]
  a. Peer‐reviewed journal article
  b. Book chapter
  c. Dissertation
  d. Conference presentation
  e. Government report, technical report, or working paper
  f. Other (specify in textbox)
4. How was this study located during the search process? [*dropdown menu*]
  a. Systematic search of electronic database
  b. Systematic search of nonacademic database
  c. Hand‐search or reference harvesting
  d. Professional contact
  e. Other (specify in textbox)
5. In what country was the intervention implemented? [*textbox*]
6. How many courts were included in the study? [*textbox*]
7. If the evaluation and/or intervention was funded, record the funding source. [*textbox*]

*SysReview allows for multiple reports of a single study to be included in the one full‐text coding record form. Each report is nested within the overall study record and the Report ID will consist of the Study ID followed by a unique alphabetical code (e.g., 1234_a, 1234_b…).

### Participants

1. Who are the participants? [*checkboxes*]
  a. Parents (mothers only)
  b. Parents (fathers only)
  c. Parents (both mothers and fathers)
  d. Other caregiver (e.g., foster parents, grandparents)
  e. Children
  f. Other (specify in textbox)
2. If applicable, describe the recruitment and sample for parent(s)/caregiver(s) using the fields below:
  a. How were participants recruited? [*textbox*]
  b. What were the eligibility criteria for inclusion in the study? [*textbox*]
  c. Describe the sample attrition. [*textboxes*]
    **Table:**
    Number of participants	Treatment	Comparison	Total
    Referred to study			
    Consented			
    Assigned			
    Began intervention			
    Completed intervention			
    Completed follow‐up 1			
    Completed follow‐up 2 (if applicable)			
  d. Describe the characteristics of the sample. [*textboxes*]
    **Table:**
    Characteristic	Treatment	Comparison	Total
    Age (mean, SD, range)			
    Gender (% female)			
    Ethnicity (proportions)			
    Socioeconomic status			
    Comorbidity			
  e. Note any other pertinent sample information (e.g., parity, marital status, education, prior criminal history, type of drug use, addiction history, or other key risk factors present). Please record for both the treatment and comparison groups [*textbox*]
3. If applicable, describe the recruitment and sample for children using the fields below:
  a. How were participants recruited? [*textbox*]
  b. What were the eligibility criteria for inclusion in the study? [*textbox*]
  c. Describe the sample attrition. [*textboxes*]
    **Table:**
    Number of participants	Treatment	Comparison	Total
    Referred to study			
    Consented			
    Assigned			
    Began intervention			
    Completed intervention			
    Completed follow‐up 1			
    Completed follow‐up 2 (if applicable)			
  d. Describe the characteristics of the sample. [*textboxes*]
    **Table:**
    Characteristic	Treatment	Comparison	Total
    Age (mean, SD, range)			
    Gender (% female)			
    Ethnicity (proportions)			
    Comorbidity			
  e. Note any other pertinent sample information (e.g., placement status, other key risk factors present). [*textbox*]
4. How was parental substance misuse substantiated? [*textbox*]
5. What type of substance misuse was captured by the study? [*dropdown menu*]
  a. Alcohol
  b. Drug (specify in textbox)
  c. Both

### General methodological details and nature of comparisons

1. What is the nature of the comparisons for this study?
  a. Single intervention contrasted with single comparison condition
  b. Multiple interventions against a single comparison condition
  c. Within one group over time
  d. Other (specify in textbox)
2. General research design classification [*dropdown menu*]
  a. Randomized controlled trial
  b. Quasi‐randomized controlled trial
  c. Nonrandomized controlled trial (e.g., interrupted time‐series, matched control group design)
  d. Other (specify in textbox)
3. What type of comparison condition was used? [*dropdown menu*]
  a. No treatment
  b. Treatment‐as‐usual (specify in textbox)
  c. Alternative treatment (specify in textbox)
  d. Waitlist control
  e. Other (specify in textbox)
4. How were treatment and comparison groups formed? [*dropdown menu*]
  a. Random allocation
  b. Matching (specify matching method and matching variables in textbox)
  c. On basis of score on a specific measure (e.g., diagnosis, specify in textbox)
  d. Self‐selection
  e. Other (specify in textbox)
  f. Unclear
5. What was the unit of allocation? [*dropdown menu*]
  a. Participant
  b. Dyads
  c. Family
  d. Service site
  e. Other (specify in textbox)
  f. Unclear
6. If participants were randomly allocated to conditions, how was this implemented? [*dropdown menu*]
  a. Simple
  b. Yoked pairs
  c. Cluster (specify cluster in textbox)
  d. Block/stratified (specify variables in textbox)
  e. Matched pairs (specify matching variables in textbox)
  f. Other (specify in textbox)
  g. Unclear
  h. Not applicable
7. Who executed the randomization? [*dropdown menu*]
  a. Researchers
  b. Practitioners
  c. Other (specify in textbox)
  d. Unclear
8. If applicable, was randomization equivalent across intervention sites? [*dropdown menu*]
  a. Yes
  b. No
  c. Unclear
  d. No applicable
9. Was group equivalence assessed?
  a. Yes (specify how this was done in textbox)
  b. No
  c. Unclear
  d. Not applicable
10. Were the treatment and comparison groups equivalent at baseline?
  a. Yes
  b. No (specify differences)
  c. Unsure
  d. Not applicable
11. Are there any differences between participants who completed versus did not complete the treatment?
  a. Yes (specify differences)
  b. No
  c. Unsure
  d. Not applicable
12. What was the unit of analysis? [*dropdown menu*]
  a. Participant
  b. Dyads
  c. Family
  d. Service site
  e. Other (specify in textbox)
  f. Unclear

### Intervention details

1. What is the name of the intervention(s), as reported by study authors? [*textbox*]
2. What settings were used during the intervention(s) (e.g., home, community, inpatient facility, school)? [*textbox*]
3. When was the intervention conducted (e.g., year)? [*textbox*]
4. Describe the intervention provided to participants. [*textbox*]
5. Describe the duration of the entire intervention. If available, describe the minimum, maximum, mean and standard deviation for intervention duration. [*textbox*]
6. Describe the intensity of the intervention (e.g., frequency of contacts and length of contacts). If available, describe the minimum, maximum, mean and standard deviation for intervention intensity. [*textbox*]
7. Who implemented the intervention? [*dropdown menu*]
  a. Nurse
  b. Social worker
  c. Psychologist
  d. Medical practitioner
  e. Other allied health practitioner
  f. Unclear
  g. Other (specify in textbox)
8. Was there more than one intervention site? [*dropdown menu*]
  a. Yes (specify number of sites in textbox)
  b. No
  c. Unclear
9. Was treatment integrity monitored? [*dropdown menu*]
  a. Yes (specify in textbox)
  b. No
  c. Unclear
10. Were there any issues with fidelity? [*dropdown menu*]
  a. Yes (specify in textbox)
  b. No
  c. Unclear
11. Did the authors report cost‐benefit data? [*dropdown menu*]
  a. Yes (specify in textbox)
  b. No
  c. Unclear

### Outcome(s) measurement*

*To be completed for each eligible outcome within a study (or group of reports for a study). To add another outcome, click the “Add another outcome” button located at the bottom of the screen.

1. What is the outcome being measured? [*textbox*]
2. What is the variable name that will be used in statistical software? [*textbox*]
3. Who does this outcome relate to? [*dropdown menu*]
  a. Parent/caregiver
  b. Child
  c. Other (specify)
4. How was the outcome measured (e.g., name of scale)? [*textbox*]
5. What are the psychometric properties of the measurement tool (e.g., reliability, validity, diagnostic thresholds, what higher /lower values mean)? [*textbox*]
6. How was the outcome data gathered? [*dropdown menu*]
  a. Self‐report
  b. Observation
  c. Official source (e.g., child protection status)
  d. Interview
  e. Other (specify in textbox)
7. Who was the respondent/participant? [*dropdown menu*]
  a. Child
  b. Parent/caregiver
  c. Teacher
  d. Practitioner
  e. Other (specify in textbox)
8. At what time‐point(s) was the outcome measured? [*textbox*]
9. Were data collected in the same manner for the treatment and comparison conditions? [*dropdown menu*]
  a. Yes
  b. No (specify in textbox)
  c. Unclear
10. Which condition does the raw difference/effect favor (ignore statistical significance)? [*dropdown menu*]
  a. Experimental condition
  b. Comparison condition
  c. Neither condition (no difference)
  d. Unclear
11. In which direction did the outcome change? [*dropdown menu*]
  a. Positive
  b. Negative
  c. Mixed (specify in textbox)
  d. Unclear
12. Were there statistically significant differences for this outcome? [*dropdown menu*]
  a. Yes
  b. No
  c. Not tested
  d. Unclear
13. What were the study author(s)’ conclusions about this outcome? [*textbox*]

### Effect size data*

*To be completed for each eligible outcome within a study (or group of reports for a study). To add another outcome, click the “Add another outcome” button located at the bottom of the screen.

1. On what page number is the effect size data reported? [*textbox*]
2. What type of effect size is being coded? [*dropdown menu*]
  a. Baseline or pre‐test measure prior to intervention)
  b. Post‐test (first point of measurement after intervention)
  c. Follow‐up (subsequent point of measurement after first post‐test)
3. What is the timeframe captured for the measure?
  a. Minimum [*textbox*]
  b. Maximum [*textbox*]
  c. Mean [*textbox*]
  d. Same for all participants (i.e., fixed) [*textbox*]
4. How was the effect size obtained for this outcome? [*dropdown menu*]
  a. Reported in document → *Go to Question 3*
  b. Calculated by user → *Go to Question 4*
5. Identify the type of effect size reported for this outcome and enter the required data for that effect size in the text boxes provided. [*textboxes*]
6. Enter the appropriate data in the relevant “Data for effect size calculations” tabs (see below). The data entered will depend on what is reported in the document. If none of the circumstances in the tabs reflect the data in the document, follow the link to David Wilson's online effect size calculator to calculate an effect size. You can enter the data in the ‘Data for effect size calculations 2′ tab in the “Other information” textbox. [*textboxes*]
